# VarSite: Disease variants and protein structure

**DOI:** 10.1002/pro.3746

**Published:** 2019-10-27

**Authors:** Roman A. Laskowski, James D. Stephenson, Ian Sillitoe, Christine A. Orengo, Janet M. Thornton

**Affiliations:** ^1^ European Molecular Biology Laboratory European Bioinformatics Institute (EMBL‐EBI) Cambridge UK; ^2^ Wellcome Trust Sanger Institute Cambridge UK; ^3^ Institute of Structural and Molecular Biology University College London London UK

**Keywords:** 3D protein structure, CATH, ClinVar, disease variants, gnomAD, molecular interactions, natural variants, PDB, Pfam, schematic diagrams, UniProt, VarMap, VarSite

## Abstract

VarSite is a web server mapping known disease‐associated variants from UniProt and ClinVar, together with natural variants from gnomAD, onto protein 3D structures in the Protein Data Bank. The analyses are primarily image‐based and provide both an overview for each human protein, as well as a report for any specific variant of interest. The information can be useful in assessing whether a given variant might be pathogenic or benign. The structural annotations for each position in the protein include protein secondary structure, interactions with ligand, metal, DNA/RNA, or other protein, and various measures of a given variant's possible impact on the protein's function. The 3D locations of the disease‐associated variants can be viewed interactively via the 3dmol.js JavaScript viewer, as well as in RasMol and PyMOL. Users can search for specific variants, or sets of variants, by providing the DNA coordinates of the base change(s) of interest. Additionally, various agglomerative analyses are given, such as the mapping of disease and natural variants onto specific Pfam or CATH domains. The server is freely accessible to all at: https://www.ebi.ac.uk/thornton-srv/databases/VarSite.

## 1 INTRODUCTION

### 1.1 Disease‐associated variants

Genetic variants can have impacts that range from benign to fatal, with a host of diseases of varying severity in between. Disease‐associated variants can occur not just in coding regions, but in regulatory regions, introns, noncoding regions, splice sites, and so on. Perhaps the best‐known source of such variants is ClinVar,^1^, ^2^ which relates DNA variants from patient samples to the associated phenotypes and their clinical significance—that is, benign or disease‐causing. It currently (June 2019) contains over 440,000 variants. Other sources of variant data include HGMD (the Human Gene Mutation Database)^3^ which collates published data on gene variants responsible for human inherited disease. Its “professional” version contains over 256,000 variants, while its public version has over 170,000. The Online Mendelian Inheritance in Man (OMIM)^4^ database, which similarly compiles genetic disorders from the literature, focuses on human genes and genetic phenotypes, and contains over 25,000 variants. UniProt^5^ stores disease variants that affect human proteins, mapping the variants onto the canonical isoform of each. The data are compiled from OMIM and ClinVar, and are made available as a downloadable file, called humsavar.txt, of over 30,000 variants.

### 1.2 Natural variants

Variants that do not cause disease, but which are present in the general population, provide a baseline measure of the natural variability in the human genome against which the disease variants can be compared. The largest source of such variants is the gnomAD database^6^ which contains data from various disease‐specific and population genetic studies of over 140,000 individuals. Currently, it holds over 15 million variants.

### 1.3 Variant impact

The severity of a novel variant, or one of clinical interest, can be assessed using one of a number of classifiers. Their predictions are based on a variety of factors, including conservation, known pathogenic mutations, and protein‐level annotations. The best known are probably SIFT (sorting intolerant from tolerant),^7^ PolyPhen,^8^ and CADD (combined annotation dependent depletion),^9^ although newer methods can give more reliable results.^10^ Various servers provide predictions on specific variants: MutPred2,^11^ MutFunc,^12^ and others. Indeed, there are over 100 tools and resources for predicting the impact of genetic variants, usefully compiled in the Variant Impact Predictor Database (VIPdb).^13^

### 1.4 Databases

A number of databases provide various levels of annotation for disease‐causing variants.^13^ One such is the DECIPHER^14^ database, which contains a large repository of genetic variants, and associated phenotypes, from an international consortium of over 200 academic clinical centers of genetic medicine, as well as over 1,600 clinical geneticists and diagnostic laboratory scientists. Although primarily focused on variants at the DNA level, the database also provides protein structural information, where the human structure is available, and a 3D viewer showing the locations of the variants in the 3D structure.

The eDGAR^15^ database contains gene/disease associations compiled from OMIM, Humsavar, and ClinVar. Its annotations come from many resources, including the Protein Data Bank (PDB),^16^ BIOGRID,^17^ STRING,^18^ KEGG,^19^ REACTOME,^20^ NET‐GE,^21^ and TRRUST.^22^

HuVarBase^23^ contains protein‐level data for disease‐causing variants and includes 3D views of the protein, when a structure of the human protein is available.

PhyreRisk^24^ overcomes the problem of missing human protein structures by generating homology models from the structures of related proteins in other organisms. The homology models are built by the Phyre2^25^ program. The protein page includes an interactive sequence browser with the variants mapped to the relevant residues, an interactive 3D view of the protein structure in the JSmol molecular viewer,^26^ highlighting the locations of the variants, a graphical image indicating how much of the protein can be mapped to a 3D structure, a list of the different isoforms of the protein in UniProt, and a list of the variants themselves. Specific variants can be further analysed using its sister server, Missense3D.^27^ This shows an assessment of the likely impact of the variant on the protein's 3D structure. It assesses the variant in its structural context and identifies possible ways in which it might affect the protein in question: for example, if the charge of a buried residues is changed, or if a disulphide bond is broken, or if the secondary structure is altered, and so on.

### 1.5 VarSite

Here we describe VarSite, a server that aggregates much of the same information as in the servers just described but adds some novel features and aims to be easier to use by the nonspecialist. It has two principal “views” of the variant data: the protein view, and the residue report. In the former, a protein's disease‐associated variants are shown in the context of the protein as a whole—both in terms of the sequence and its various properties (i.e., Pfam domains, residue conservation, natural variants, structural annotations, etc.), and the regions where at least one 3D structure is available. The structural information comes from all closely related proteins in the PDB—both human and non‐human. This provides a far greater coverage, and more information, than is given by many of the other servers which map only to a structure of the exact human protein itself. The second principal view is the residue report which analyses a given variant in detail. This information can help assess potential pathogenicity. The analysis takes in the magnitude of the change in amino acid properties, the disease propensity of the change, and its structural context. One novel feature is VarSite's analysis by domain (Pfam or CATH), wherein it aggregates variants taken from human proteins containing the given domain and provides an indication of whether the domain may contain “hot spots” for disease‐associated variants. Another novel feature is the use of the Disease Ontology to map which parts of the body are affected by the specific disease.

Thus, VarSite aggregates and extends annotations from various resources, provides various “views” on the data, and offers new functionality in order to further facilitate the interpretation of variants,

## 2 PROTEIN VIEW

### 2.1 Variants

In the protein view, each human protein in UniProt has its own VarSite page, with the variant and structural data mapped onto a schematic representation of the protein sequence. At present (June 2019), only the canonical isoforms from UniProt are included. These are the sequences to which the variant data in UniProt are mapped. However, it is planned to extend VarSite to eventually include all other isoforms.

The variants come in three types: disease‐associated, which come with a disease identifier (e.g., CF for cystic fibrosis); “disease notes,” which have no disease identifier, being merely remarks on some consequence of the variant (e.g., “reduces biological activity”); and mutagenesis experiments where the consequence of an engineered variant is given (e.g., “loss of DNA binding”). Throughout VarSite, these variants are color‐coded: red for disease‐associated, gray for disease notes, and green for mutagenesis experiments.

Only variants in coding regions are included, as these can be mapped first onto the protein sequence, and thence onto a 3D structure, if available. They are primarily missense variants where one amino acid is changed to another, but also include premature stop codons, stop loss, start loss, deletions, insertions, and sequence replacements. Excluded are variants that result in a frame shift that disrupts the protein's sequence, and cancer‐causing somatic variants. The latter would swamp the germline data and complicate the analyses.

In addition to the variants obtained from UniProt, we include those obtained from ClinVar. However, as these are in DNA coordinates (i.e., chromosome, location, and base change), they need to be translated to the corresponding protein sequence position in the canonical UniProt isoform. We use our VarMap^28^ server to perform this translation on all the ClinVar data before being able to incorporate them into VarSite. VarMap performs the mapping using a number of tools and databases, including Ensembl,^29^ VEP,^30^ UniProt, and BioMart.^31^ Some ClinVar data are already present in UniProt, so duplicates are identified via their reference “rs” identifier and removed.

A summary at the top of each VarSite protein page shows the numbers of each of the three types of variant, together with general information about the protein, obtained from UniProt, such as is function, tissue specificity, and sequence length.

### 2.2 Associated disease(s)

**Figure 1:**
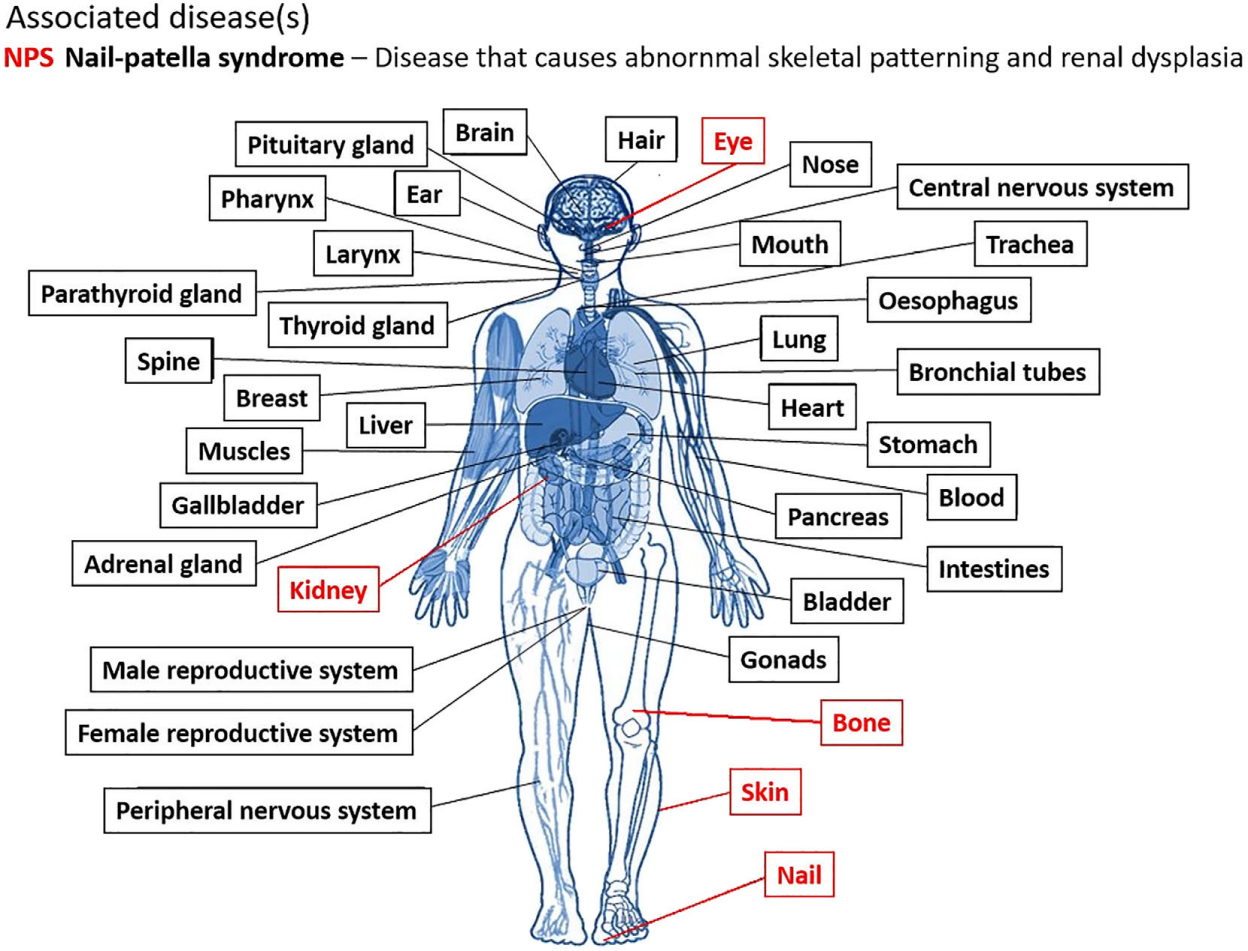
Disease association for variants in the LIM homeobox transcription factor 1‐beta protein (UniProt accession O60663). List of diseases—in this case just nail‐patella syndrome—and a schematic diagram of the affected organs (highlighted in red): eyes, kidneys, bones, skin, and nails. Clicking on any of these opens up the organ's Disease Ontology page

Next, come a list of any diseases associated with the protein. Figure 1 shows the disease associated with the example protein we will be using in this article: LIM homeobox transcription factor 1‐beta (UniProt accession O60663). This protein plays a central role in the dorsoventral patterning of vertebrate limbs, and is associated with nail‐patella syndrome^32^ (disease id NPS; OMIM reference: 161200)—an inherited developmental disorder that most often results in abnormalities of the nails, knees, elbows, and pelvis, and can also affect the eyes and kidneys. The red labels in the figure highlight the organs affected by the disease. Clicking on any of them takes you to that organ's entry in the disease ontology. The ontology has been derived by mapping the diseases defined in UniProt to the Monarch Disease Ontology.^33^

### 2.3 Protein and variants

**Figure 2:**
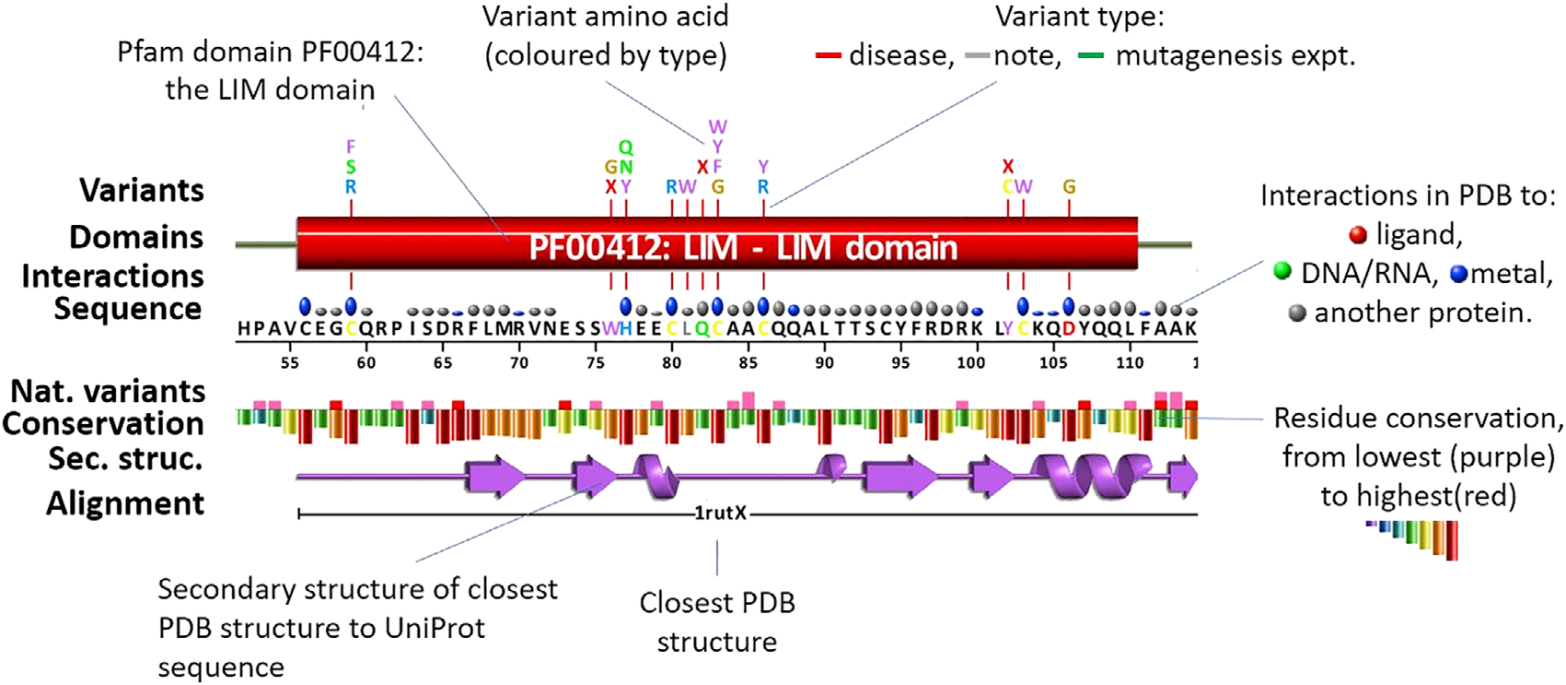
A schematic VarSite diagram of part of the LIM homeobox transcription factor 1‐beta protein (UniProt accession O60663), with its various sequence and structural annotations. Shown are residues 52–114 (of 402), containing a LIM domain (red cylinder), as defined by Pfam. The disease‐associated variants are shown by their single‐letter amino acid codes above the domain diagram, colored by residue type (blue, positive; red, negative; green, neutral; gray, aliphatic; purple, aromatic; brown, proline, and glycine; yellow, cysteine). The disease in question is nail‐patella syndrome, which causes abnormal skeletal patterning and renal dysplasia. Above the line giving the protein's sequence and residue numbering is a line of colored blobs of different sizes. These correspond to the types and numbers of intermolecular interactions made by the equivalent residues in the associated 3D structures in the PDB. The larger the blob, the more structures the interaction occurs in. The color of the blob indicates the type of interaction that dominates: metal (blue), ligand (red), DNA/RNA (green), and protein–protein (gray). The “best” PDB entry (PDB code 1rut, chain X), in terms of closest sequence identity and structural quality, is shown schematically in the purple “wiring diagram,” where beta strands are represented by arrows, and alpha helices by coils. Above it is a graph of residue conservation, computed from a sequence alignment obtained from a BLAST search against the UniProt database. The bars are colored from red for highly conserved to purple for highly variable. One noticeable feature is that the residues that interact with metals (marked by the blue blobs) tend to be highly conserved, and also tend to be the ones having disease‐associated variants. The graph above the conservation plot shows where natural variants have been identified in this protein. The data come from gnomAD, and the bars are colored according to the normalized CADD scores of the variants (red, CADD>30; pink, CADD>25; orange, CADD>20; green, otherwise). Clicking on the various annotations gives a pop‐up window with further information, some examples of which are shown in Figure 3

The key part of the protein page is the schematic diagram illustrating where on the protein the various variants occur. The diagram includes structural (and other) annotations. Figure 2 shows part of our example protein, namely the region encompassing the first of its two LIM domains. A LIM domain is defined in Pfam^34^ as “composed of two contiguous zinc finger domains, separated by a two‐amino acid residue hydrophobic linker” (Pfam id PF00412). The LIM domains mediate protein–protein interactions critical to cellular processes. Their main component, the zinc finger, is a structural motif in which a zinc ion is coordinated by four of the protein's sidechains, typically Cys or His, to stabilize the fold. Loss of one or more of these sidechains, say by mutation to a different residue type, will disrupt the fold and likely disable or hamper the protein's biological function.

**Figure 3:**
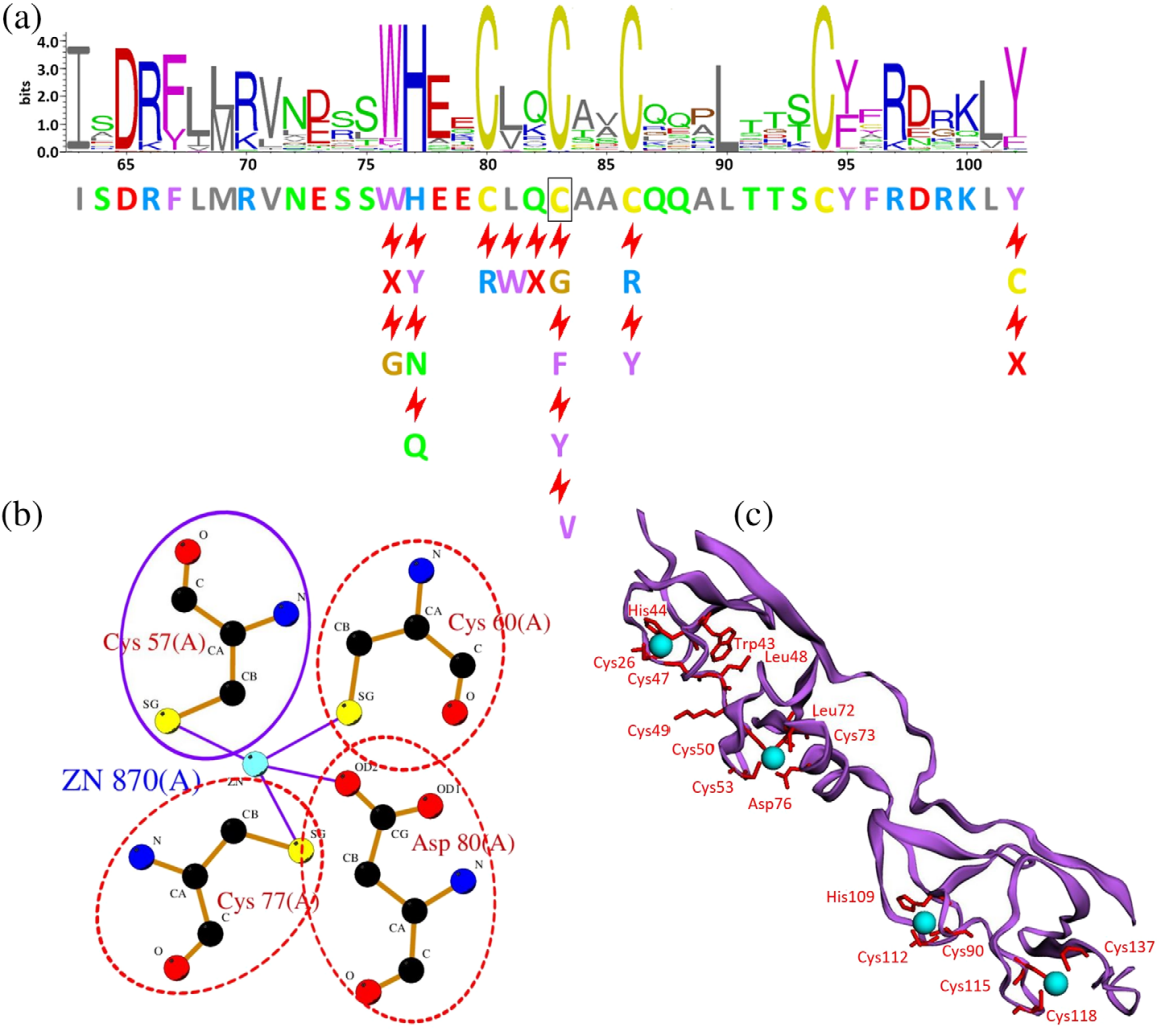
Example analyses obtained by clicking on the annotations in the schematic diagram shown in Figure 2. (a) Forty‐residue sequence logo centred on Cys83, showing the residue conservation at each position from a multiple alignment of homologous proteins. The alignment was obtained from the pairwise alignments returned by a BLAST search of our protein (UniProt accession O60663) against all UniProt sequences. The taller the letter, the more commonly it is found at that position in the alignment. The colors of the amino acids are as described in the legend to Figure 2. Residues marked by the red lightning bolts are the known disease‐associated variants at those sequence positions. An “X” corresponds to cases where a base change has resulted in a stop codon, and hence truncated protein at that position. (b) A schematic diagram of the interactions the residue (circled in purple) makes with a zinc ion, this time in PDB entry 3mmk. Other disease‐associated residues are circled by red dotted ovals. (c) A 3D view of the closest PDB entry (code 1rut, chain X). The protein's secondary structure is shown in purple, with the disease‐associated residues shown as red sidechains and labeled in red. The cyan spheres are zinc ions held in place by four coordinating Cys or His sidechains. The structure is rendered by the 3Dmol.js viewer

In Figure 2, the LIM domain is depicted by the red cylinder. Above it are shown the disease‐associated variants from UniProt and ClinVar, their single‐letter amino acid codes colored by residue type. Some residues have more than one disease‐associated variant, most notably Cys83, which has four. The red bar in the residue conservation plot shows the Cys is very highly conserved which implicates it as critically important. Other disease variants also tend to be of highly conserved residues, as one might expect. Clicking on any of the conservation bars pops up a “sequence logo,” as shown in Figure 3a, which not only shows which are the most highly conserved residues, but also the range of residue types that are found at each position. Residue conservation is based on sequence alignments obtained from a BLAST^35^ search of our sequence against all the sequences in UniProt. The conservation score is computed using the ScoreCons^36^ method.

### 2.4 Natural variants

Above the conservation plot in Figure 2 is a plot representing the natural variants in this protein, as obtained from gnomAD. The bars are colored according to the CADD scores of the variants: red, for severe consequence, through pink and orange, to green for benign. Like the ClinVar data, the gnomAD variants relate to genomic coordinates and have to be converted to the canonical UniProt isoform sequence positions using VarMap.

It is interesting to note how the disease and natural variants in Figure 2 tend to avoid the same residues. This will not be the case for all proteins as the gnomAD data are not limited to healthy individuals, and some genetic diseases do not reveal themselves until late in life.

### 2.5 Structural annotations

Also shown in Figure 2 are various structural annotations obtained from homologous proteins of known 3D structure in the PDB. The structural data come from PDBsum.^37^ Proteins are considered homologous if the FASTA^38^ sequence search returns an *E*‐value below 10^−3^, *z*‐score above 4.0, and sequence identity greater than 20%. In Figure 2, the secondary structure of the closest structure—PDB code 1rut, chain X—is shown schematically in purple, below the residue conservation plot. Clicking on the secondary structure will pop up a window showing the 3D structure itself in the 3Dmol.js^39^ interactive viewer, as shown in Figure 3c. Here, the protein's secondary is shown in purple cartoon, while the disease‐associated variants are depicted by labeled, red sidechains. As is often the case, the numbering of the residues in the PDB file differs from the UniProt sequence numbering; the equivalences are obtained from the FASTA alignment and are shown in the pop‐up window. Depending on how close the PDB sequence is to that of the UniProt protein, one or more residues in the PDB file may actually be of a different residue type—for example, a Cys in the UniProt sequence may be, say, an Ala in the PDB structure.

The PDB structures can provide valuable information on which residues interact with other molecules: ligands, metal ions, DNA/RNA, or other proteins. Such interacting residues may be important for the protein's function. However, PDB structures are a mixed bag. Some may be of a complex with a ligand or DNA fragment, while others may have nothing bound. Some may have missing residues, others may be missing entire domains. Thus, selecting a single, representative structure, may miss such information, depending on which structure is chosen. To reap the full benefit of the information in the PDB requires taking into account interaction data from all homologous 3D structures, not just one. The line of colored blobs in Figure 2, labeled “Interactions” summarizes these data. The blobs are colored by the most common type of interaction the given residue is involved in: metal (blue), ligand (red), DNA/RNA (green), and protein–protein (gray). The larger the blob, the greater the percentage of structures having that type of interaction. Clicking on any of the blobs opens a pop‐up window providing more information about the residue's interactions, and the full list of the homologous proteins in which it occurs. Links lead to schematic diagrams of the interactions, such as the LIGPLOT^40^ diagram of metal interactions for Cys83 shown in Figure 3b. The Interactions line also highlights catalytic residues and disulphide bonds, where present.

### 2.6 Variants by domain

Protein domains often contain residues that are vital either for maintaining the domain's stability, or for performing its biological function. Any change affecting these residues is likely to disrupt the protein and/or its function and lead to a pathological phenotype. Identifying such crucial residues can be difficult for some proteins—perhaps because there is no corresponding 3D structure, or because few, or no, disease‐associated variants have been identified to date. However, by aggregating the variant data for a given domain from all the proteins that contain it, one might be able to observe which residues positions are commonly associated with disease.

VarSite uses two types of domain definitions: the sequence‐based domains of Pfam, and those based on 3D structure from CATH functional families^41^ (FunFams). These are a subclassification of the CATH domain definitions.^42^ The members of each FunFam are likely to share highly similar structures and functions. Consequently, the structure‐based sequence alignments obtained give a more reliable mapping of equivalent residues in the domains from different protein.

### 2.7 Other information

The protein view also includes various other links, where relevant. For example, a list of the most “informative” interacting ligands is given, based on the information in the homologous structures. Most “informative” are those which, for example, match the protein's known substrate, or are drugs known to target the protein, or interact with one or more disease‐related residues, or just occur more frequently in the structures.

Links to reaction pathways are also given, when known, being the pathways defined by UniProt, and those given in the REACTOME.^20^

For variants obtained from UniProt, the literature citations for each variant, together with links, are listed at the end of the page.

## 3 RESIDUE REPORT

The second main “view” VarSite provides onto variant data is the Residue Report. This gives various structure‐based, and other, analyses of a specific amino acid variant to help assess whether it might be benign or deleterious.

### 3.1 Residue change

**Figure 4:**
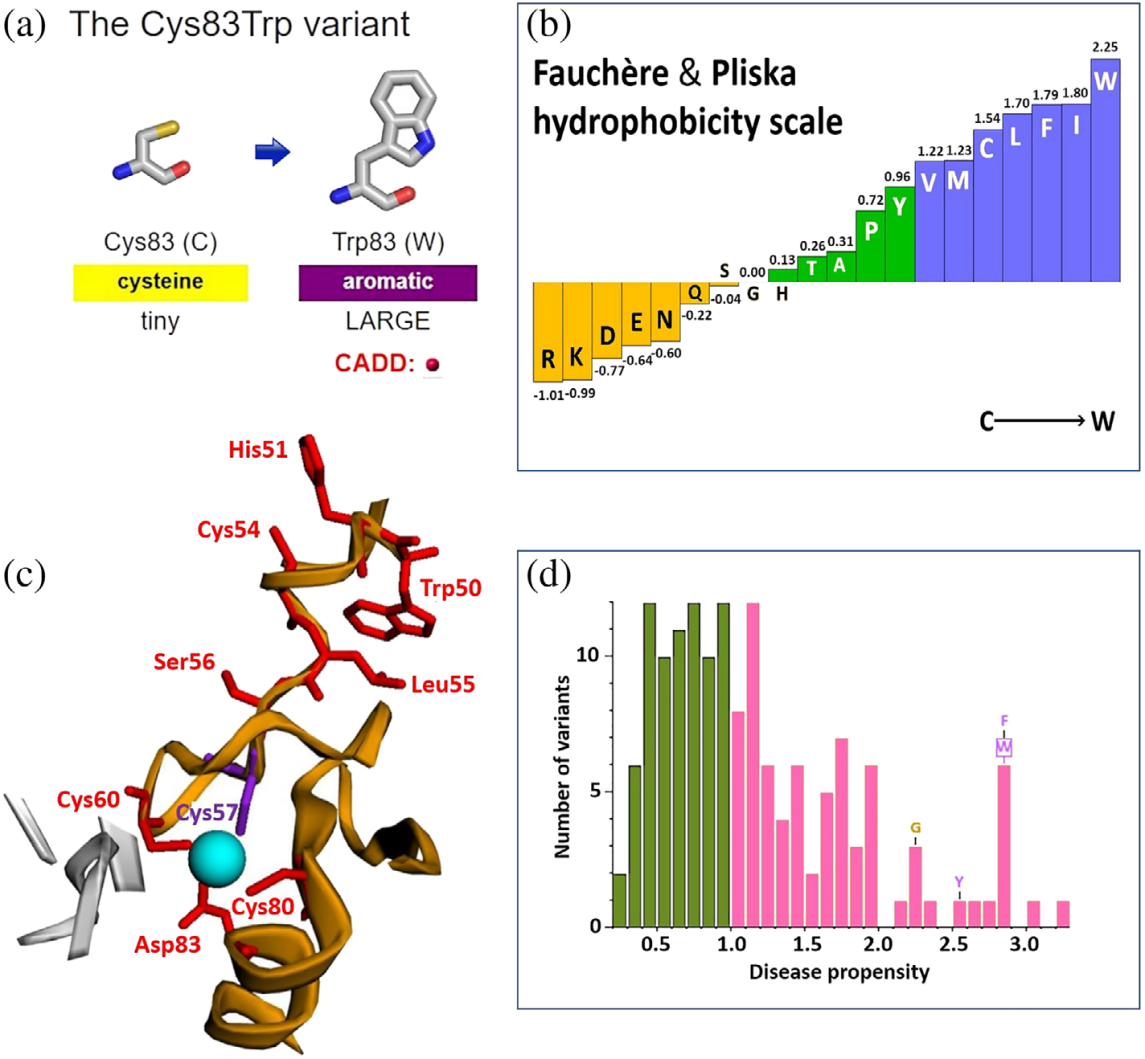
Some of the analyses given in the residue report for variant Cys83Trp in UniProt entry O60663. (a) A schematic diagram of the variant itself, listing the properties of each sidechain involved. Here we see a tiny cysteine sidechain going to a large, aromatic residue—a change having an unfavorable normalized CADD score (indicated by the red dot). (b) The change from Cys to Trp is indicated on the Fauchère and Pliska hydrophobicity scale, showing that, in terms of hydrophobicity, the change is not too severe. (c) A close‐up of the variant's location in a closely related protein 3D structure (PDB code 2ypa, Chain C). The variant residue is shown in purple sticks and labeled Cys57—which is the equivalent residue in this structure. (d) A histogram of the disease propensity scores of all possible *aa*_1_ → *aa*_2_ amino acid changes. The locations on this plot of the four disease‐associated variants of Cys83 are labeled, with our Cys → Trp variant highlighted by a box. Disease propensities above 1.0 are colored pink, while the more likely benign changes, scoring below 1.0, are colored green. The disease propensity of Cys → Trp is 2.84

Figure 4 shows some of the analyses from the report for Cys83Trp in our protein, UniProt entry O60663. Figure 4a shows the original and mutated sidechains, and the corresponding change in physicochemical properties: a tiny cysteine sidechain being replaced by a large, aromatic tryptophan residue. The deleteriousness of the change is gauged by the commonly used normalized CADD score, indicated by the red dot, which in this case is a high 28.9. CADD scores are derived from more than 60 genomic features and are computed using a machine learning model. The precomputed scores for every possible variant at every position in the human genome can be downloaded. These are in genomic coordinates, so, like the ClinVar and gnomAD data, need to be mapped onto the corresponding canonical UniProt isoform sequences using the VarMap utility.

### 3.2 Summary report

A bullet‐pointed summary appears at the top of the Residue Report and color‐codes different aspects of the variant in question. Points highlighted in red are likely to be deleterious, in orange less so, and in green benign. Further details on each property can be found in the report itself.

### 3.3 Change in hydrophobicity

The histogram in Figure 4b shows the relative hydrophobicities of the 20 amino acids, according to the Fauchère and Pliska hydrophobicity scale.^43^ The change corresponding to the variant: C → W is marked on it, indicating the change is not a large one, as both residues are rather hydrophobic.

### 3.4 Disease propensity

Figure 4d shows the distribution of observed “disease propensities” and where our C → W variant occurs (boxed). Also labeled are this residue's other disease‐associated variants, C → G, Y, and F.

The disease propensity is a simple, normalized ratio of the number of disease‐to‐natural variants of a given type (e.g., *Ala*→*Phe*). The disease variant counts come from UniProt and variants in ClinVar annotated as deleterious, while the natural variant counts come from gnomAD. The highest propensity is 3.27 for C → R, meaning that C → R variants are found more than three times as often in the disease data as in the natural data. The lowest disease propensity is 0.25 for I → V.

### 3.5 3D structure and interactions

Figure 4c shows the location of the variant in the closest, and “most informative” protein 3D structure (PDB code 2ypa, Chain C), displayed using the 3Dmol.js application. Note that, this is a different PDB structure from that used in Figure 3b and focuses on a region of 12 Å around the variant residue. The structure that is chosen here depends on which has the most informative features within the 12 Å radius: namely, most disease‐associated residues, most interactions with another molecule (DNA, metal, ligand, etc.), and the closest in sequence identity to our protein. As before, the residue numbering in the PDB entry may be different from the UniProt sequence; here the residue corresponding to Cys83 is Cys57(C) in the PDB structure and is colored purple. Indeed, not only might the residue numbering differ, but sometimes the residue in the PDB structure might be a different residue altogether.

## 4 USER‐SUBMITTED VARIANTS

Users interested in a specific variant, or set of variants, can use the Find Variant form on the VarSite home page to locate them and get an individual Residue Report for each. Three types of input are accepted:

1. UniProt accession, sequence position, residue type, and variant type—for example, P19544, Cys355Arg.
2. Genomic location (chromosome number and coordinate), base change, and genome build (Chr.37 or Chr.38)—for example, chromosome 3, genomic coordinate 44,448,385, base change T → C, Chr.38.
3. A tab‐separated list of DNA coordinates, as in Option 2 above. Again, the build number is required, although if there are more than 20 entries in the list, the scripts will detect which is the more appropriate build: Chr.37 or Chr.38.

The search uses the same procedure, and same scripts, as VarMap and its outputs are the same. Each coordinate that occurs in a protein coding region has a link to the Residue Report for the corresponding amino acid variant.

The search is performed in real time except where more than 20 coordinates are entered in Option 3. In this case, the user's e‐mail address is requested, and the search is performed in batch mode, in parallel on the EBI processor farm. A link to the results is e‐mailed to the user when all the searches are complete.

## CONFLICT OF INTEREST

None declared.

## Data Availability

VarSite is freely available at https://www.ebi.ac.uk/thornton-srv/databases/VarSite.
